# Is it possible to estimate the number of patients with COVID-19 admitted to intensive care units and general wards using clinical and telemedicine data?

**DOI:** 10.31744/einstein_journal/2024AO0328

**Published:** 2024-02-23

**Authors:** Caio Querino Gabaldi, Adriana Serra Cypriano, Carlos Henrique Sartorato Pedrotti, Daniel Tavares Malheiro, Claudia Regina Laselva, Miguel Cendoroglo, Vanessa Damazio Teich

**Affiliations:** 1 Hospital Israelita Albert Einstein São Paulo SP Brazil Hospital Israelita Albert Einstein, São Paulo, SP, Brazil.

**Keywords:** COVID-19, Coronavirus infections, Pandemics, Forecasting, Telemedicine, Resource allocation, Decision support systems, clinical, Big Data

## Abstract

**Objective:**

To develop and validate predictive models to estimate the number of COVID-19 patients hospitalized in the intensive care units and general wards of a private not-for-profit hospital in São Paulo, Brazil.

**Methods:**

Two main models were developed. The first model calculated hospital occupation as the difference between predicted COVID-19 patient admissions, transfers between departments, and discharges, estimating admissions based on their weekly moving averages, segmented by general wards and intensive care units. Patient discharge predictions were based on a length of stay predictive model, assessing the clinical characteristics of patients hospitalized with COVID-19, including age group and usage of mechanical ventilation devices. The second model estimated hospital occupation based on the correlation with the number of telemedicine visits by patients diagnosed with COVID-19, utilizing correlational analysis to define the lag that maximized the correlation between the studied series. Both models were monitored for 365 days, from May 20^th^, 2021, to May 20^th^, 2022.

**Results:**

The first model predicted the number of hospitalized patients by department within an interval of up to 14 days. The second model estimated the total number of hospitalized patients for the following 8 days, considering calls attended by *Hospital Israelita Albert Einstein’s* telemedicine department. Considering the average daily predicted values for the intensive care unit and general ward across a forecast horizon of 8 days, as limited by the second model, the first and second models obtained R² values of 0.900 and 0.996, respectively and mean absolute errors of 8.885 and 2.524 beds, respectively. The performances of both models were monitored using the mean error, mean absolute error, and root mean squared error as a function of the forecast horizon in days.

**Conclusion:**

The model based on telemedicine use was the most accurate in the current analysis and was used to estimate COVID-19 hospital occupancy 8 days in advance, validating predictions of this nature in similar clinical contexts. The results encourage the expansion of this method to other pathologies, aiming to guarantee the standards of hospital care and conscious consumption of resources.

## In Brief

Gabaldi et al. utilized telemedicine data, web search trends, hospitalized patient characteristics, and resource usage data to estimate bed occupancy during the COVID-19 pandemic. The results showcase the potential of data-driven strategies to enhance resource allocation decisions for an effective pandemic response.

## Highlights

▪ Developed models to forecast bed occupancy for up to 14 days and monitored errors for 365 days.
▪ Telemedicine calls from COVID-19 patients correlated with the number of patients hospitalized in the next 8 days.

## Graphical abstract

**Figure:**
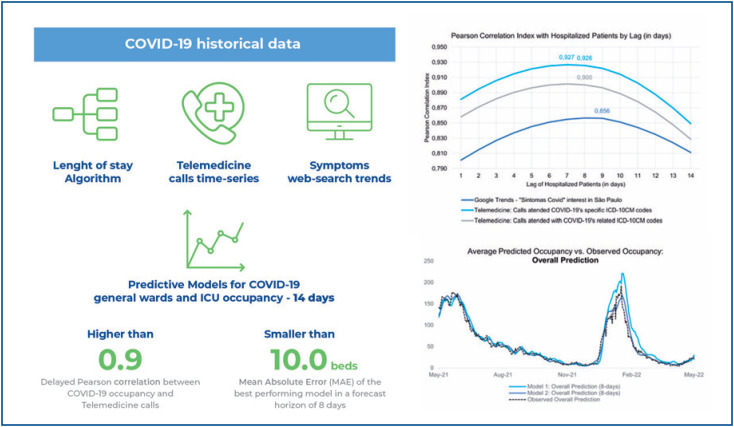

## INTRODUCTION

COVID-19 transmission rates in Brazil sustain a scenario that can be classified as a pandemic state, and the country faces waves of variable demands for physical and human resources to treat patients with COVID-19.^(1,2)^ Meanwhile, the priority of health service managers and the government^(3)^ has been to provide proper sizing of its resources to guarantee the access of the population to appropriate care. It is necessary to ensure that patients, at all severity levels, who seek assistance during the pandemic have access to supplies, medicines, equipment, beds, and medical staff appropriate to their needs;^(4)^ aiming to improve their outcomes and minimize the negative impacts of the pandemic.

In this context, initiatives to support the decision-making process regarding demand predictability and resource sizing have become more relevant as Brazil emerged as the epicenter of the COVID-19 pandemic.^(5)^ Planning a volume-based resource allocation is an optimization alternative,^(6)^ considering that the demand for experienced intensive care unit professionals, ventilation and monitoring devices, drugs used for tracheal intubation protocols, and hospital beds has varied significantly since the beginning of the pandemic, competing with other demands for hospital care.

An increasing application of machine learning techniques has been observed in datasets containing patients’ clinical variables to predict the degradation of clinical conditions, mortality, and length of stay (LOS),^(7-9)^ as well as an approach utilizing time-series analysis to estimate hospitalizations and admissions in health institutions using the number of telemedicine visits^(10)^ or interest in terms related to pathology symptoms in web search engines (*e.g.*, Google Trends).^(11)^

This context highlights the opportunity to develop statistical models to predict the number of patients hospitalized due to COVID-19 and help hospital managers plan for beds, human resources, and other input sizing and availabilities.

## OBJECTIVE

To develop, implement, and monitor a predictive model to estimate the number of patients hospitalized due to COVID-19, segmenting patients by department-intensive care units and general wards-whenever possible.

## METHODS

This retrospective study was conducted at *Hospital Israelita Albert Einstein* (HIAE), a private not-for-profit 624-bed hospital in São Paulo, Brazil, which earmarked 300 beds for COVID-19 patients in March 2021, at the peak of the pandemic.

Two separate models were developed to achieve the study objectives:

Model 1: Hospital occupancy was estimated by projecting the hospital admissions and discharges of COVID-19 patients separately.

Hospital admissions were estimated separately for the general ward and intensive care unit (ICU), including semi-intensive care beds, and analyzed using a time-series approach, including the evaluation of seasonality, trends, and residues.

To capture the internal transfer behavior that relocated patients between the general ward and the ICU, an estimation was also proposed considering the rate of patients transferred from the general ward to the ICU as a function of LOS, considering data from the previous 30 days.

Discharges for the 14 subsequent days were estimated using an individual LOS predictive model. To build this model, the first stage consisted of a retrospective investigation of the existing correlations between clinical variables and patients’ LOS. Data were extracted from the medical charts of 4,741 patients admitted to the hospital with COVID-19 between March 2020 and July 2021.

Patients with a total LOS >45 days were excluded from the analysis because of the low representativeness of the group (<5% of the study population), and the possibility of reallocating those patients to non-COVID beds during their hospitalization, considering the clinical assessment, testing, and criteria established by global health institutions.

Additionally, for the estimation of LOS, patients who died during hospitalization, those who were transferred to another hospital, or whose discharge was requested by the patient were excluded from the analysis to isolate a pattern of behavior from observed hospitalizations and eliminate confounding factors. Firstly, feeding the model with patients who died, opted for home-care treatment, or transferred to another hospital may distort the expected impact for severity indicators such as the ventilation device use or ICU bed occupancy, resulting in an increase in the expected LOS in cases of regular discharge. Secondly, death, home care, and transfers to other hospitals represented >5% of the total number of COVID-19 hospitalizations in HIAE.

The clinical variables considered and analyzed for the LOS model included age group, sex, use of an extracorporeal membrane oxygenation (ECMO) device during the stay, use of non-invasive and invasive ventilation devices in the last 24 hours, occupancy of ICU or semi-ICU in the previous 24 hours, prescription of vasoactive drugs, and prescription of hemodialysis, with the considered criteria for inclusion detailed in the results section.

To facilitate data comprehension, an initial exploratory univariate analysis was performed, calculating the median and mean LOS of discharged patients., Comparisons between groups were carried out using the Mann-Whitney U test^(12)^ and the Kruskal-Wallis test.^(13)^ Additionally, as a multivariate approach, the impact of daily changes registered in the selected set of variables was evaluated using a random forest algorithm to dynamically predict the LOS for each individual, where the most important features were ranked and selected using the Gini importance index.^(14)^

Model 2: The correlation between the number of telemedicine visits related to COVID-19 symptoms, the volume of web searches for terms associated with COVID-19 originating in São Paulo, Brazil, and the number of patients hospitalized with COVID-19 at HIAE were evaluated.

The data used to build the second model were collected from three sources: i) the daily volume of searches in Google for the term "*sintomas* COVID-19" in São Paulo, Brazil, extracted from Google Trends-a public database containing a normalized interest score calculated based on the search volume for a given search term and location over a selected period; ii) A dataset containing the daily number of visits to the telemedicine department of HIAE, considering three distinct groupings: Group 1, patients assigned diagnosis codes related to COVID-19 (ICD-10-CM from chapter X: J00-J99, Diseases of the respiratory system; chapter XXI: Z00-Z99, Factors influencing health status and contact with health services; COVID-19’ specific ICD-10-CM code U07.1; and ICD-10-CM codes related to its symptoms: R05, R06, R07.0 and R50); Group 2: patients exclusively assigned the COVID-19-specific ICD-10-CM code U07.1; and, Group 3: the daily number of visits to the telemedicine department, without filtering ICD-10-CM codes; iii) A dataset containing the target variables: daily number of admissions and patients hospitalized due to COVID-19 at HIAE, considering only the coronavirus specific ICD-10-CM code B34.2.

At the time of data collection, the telemedicine department still did not assign the COVID-19 specific ICD-10 CM code B34.2 to identify confirmed cases of COVID-19 in its calls; therefore, this specific code was not considered in the development of the model.

The Pearson Correlation between telemedicine attendance volumes, Google Trends interest scores, and the daily normalized weekly moving averages of hospitalized patients was calculated to investigate the lag that maximized the correlation function between the number of hospitalized patients and the given series, using a regression model.^(15)^

Performance metrics: performance monitoring of the proposed models employed accuracy metrics of point forecasts-mean error (ME), mean absolute error (MAE), and root mean squared error (RMSE) as a function of the forecast horizon in days. Considering the average daily predicted values for the ICU and general ward across a forecast horizon, R² was also calculated to demonstrate the adherence of the projected values to the observed values.^(16)^

### Confidentiality and ethical approval

This study was approved by the Ethics Committee of *Hospital Israelita Albert Einstein* (HIAE), CAAE: 51937121.6.0000.0071; # 5.136.309. Patient confidentiality was preserved by anonymizing the medical records. The requirement for informed consent was waived by the institutional review board prior to data collection and analysis.

## RESULTS

Model 1: between March 2020 and July 2021, 5,414 patients were admitted to HIAE due to COVID-19, considering all types of discharges (home care, external transfers, and deaths, which were excluded from LOS modeling) and patients who were hospitalized for more than 45 days.

Admissions: by analyzing the behavior of the overall number of new hospitalizations during the specified period, segmentation of the number of new admissions by department (general ward and ICU) was proposed to minimize the error of the expected flow for each projected day, attempting to extract seasonal factors and decrease the order of magnitude of the residues.

During the analyzed period, the average (± standard deviation) number of daily patient admissions in the general ward was 7.74 (± 4.66), while new hospitalizations in the ICU was 2.85 (± 2.08).

Based on the decomposition of the time series of admissions, a seasonal component was identified for admissions in the general ward by day of the week, registering lower volumes during weekends (Table 1) and, considering the presenting characteristics, the naïve estimation of the number of admissions in the general ward included the seasonal components and the moving average of hospitalizations in the seven previous days. No seasonal patterns were identified for ICU admissions; attaching this fact to the low magnitude of average daily hospitalizations, the study modeled the daily ICU hospitalizations exclusively with its weekly moving average.

**Table 1:** Seasonal factors calculated after analyzing the behavior of admissions to general wards per day of the week

Day of the week	Seasonality factor calculated for general ward admissions
Monday	1.10
Tuesday	1.02
Wednesday	1.00
Thursday	1.04
Friday	1.05
Saturday	0.89
Sunday	0.90

Transfers: transfers between departments were estimated using a population model, considering the rate of patients transferred by the day of hospitalization since admission, calculated for 30 days prior to the projection date.

Discharges: between March 2020 and July 2021, 4,741 COVID-19 patients were discharged from HIAE after applying the exclusion criteria detailed in the Methods section to reduce the confounding factors. The selected group presented an average LOS of 8.59 days with a standard deviation of 5.78 days. A Shapiro-Wilk normality test showed with 95% confidence that the data were not normally distributed, directing the modeling of the phenomenon of interest to nonlinear strategies.

Variables that could impact LOS were discussed with ICU physicians, and their impact was evaluated by calculating the average LOS segmented by epidemiological characteristics (sex and age ranges) and severity, proxied by the use of supportive ventilation, prescription of ECMO, hemodialysis, vasoactive drugs, and ICU occupancy. For the ICU analysis, the separate impact of hospitalization in an ICU or semi-ICU on the previous day was evaluated.

The significance of the difference in LOS between the groups was tested using the Mann-Whitney U test with a 95% confidence interval. For age group stratification, the Kruskal-Wallis test was applied, followed by the Dunn test, which compared the groups individually. The proposed univariate approximation estimated the predictive potential of the subsequent inclusion of variables in the model. The results are summarized in table 2.

**Table 2:** Descriptive analysis of COVID-19 patients’ length of stay segmented by epidemiological characteristics, resource use, and predictors of severity

		Length of stay						Kruskal-Wallis Test	
		Frequency	(%)	Mean	Standard Deviation	Median	Interquartile range	χ^2^	p value
Age group, years									
	≤39	833	17.57	6.46	4.60	5	5	446.52	<0.05
	40-59	2055	43.35	8.23	5.31	7	6		
	60-79	1378	29.07	10.77	6.63	9	8		
	≥80	475	10.02	12.11	7.18	11	10		
								Mann-Whitney U Test	
Sex								W	p value
	Female	1707	36.01	8.85	6.30	7	8	2743049	<0.05
	Male	3034	63.99	9.15	5.97	7	7		
ICU bed occupancy									
	No	3700	78.04	7.36	4.60	6	5	644273	<0.05
	Yes	1041	21.96	15.04	6.91	14	10		
Semi-ICU bed occupancy									
	No	3203	67.56	7.01	4.62	6	5	956309	<0.05
	Yes	1538	32.44	13.29	6.59	12	9		
Usage of invasive mechanical ventilation device									
	No	4233	89.28	7.93	5.00	7	6	222472	<0.05
	Yes	508	10.72	18.31	6.51	18	10		
Usage of non-invasive mechanical ventilation device									
	No	2700	56.95	6.25	4.02	5	4	909496	<0.05
	Yes	2041	43.05	12.75	6.39	11	8		
Prescription of vasoactive drugs									
	No	4525	95.44	8.93	5.89	7	6	461625	0.16
	Yes	216	4.56	11.54	9.09	10	8		
Usage of ECMO therapy									
	No	4725	99.66	9.01	6.06	7	7	11450	<0.05
	Yes	16	0.34	18.88	7.82	19.5	16		
Prescription of hemodialysis									
	No	4621	97.47	8.80	5.85	7	7	83452	<0.05
	Yes	120	2.53	18.63	7.39	18	12		

ICU: intensive care unit; ECMO: extracorporeal membrane oxygenation.

Age, sex, use of ICU and semi-ICU, and use of non-invasive and invasive ventilation, including ECMO, were statistically correlated with patients’ LOS.

The prescription of ECMO was discarded while training the model because, although it indicated hospitalizations of greater complexity and severity, it was a rare intervention, observed in only 0.34% of the sample, and the group of patients with an ECMO prescription showed the highest variability among the analyzed groups, represented by its interquartile range.

A multivariate approach was proposed with a random forest regression algorithm intended to dynamically estimate the LOS for each patient on a daily basis, evaluating the impact of the class of bed occupied (ICU, semi-ICU, or general ward), adopted treatments, and specific medication prescription (mechanical ventilation devices, among others) in the last 24 hours alongside the personal characteristics of each patient, such as sex and age group.

The final step regarding the selection of available variables was performed alongside the model training routine, setting a cutoff for the Gini Importance Index, also known as the Mean Decrease in Impurity, to evaluate the feature relevance in a multivariate context. The variables included in the LOS predictive model had Gini importance indices >0.001, as presented in Table 3.

**Table 3:** List of variables tested in the development of the random forest algorithm, along with their respective Gini importance indices

Gini Index	Feature
0.6862433	Length of stay (Total time spent in ICU and Emergency departments at the moment of prediction, in days)
0.1572006	Occupied an ICU bed in the last 24 hours
0.0691286	Occupied a semi-ICU bed in the last 24 hours
0.0274035	Use of a mechanical ventilation device in the last 24 hours
0.0177144	Age group, 60-79 years
0.0129082	Age group, ≥80 years
0.0101150	Maximum axillary temperature ≥38.5 ºC in the last 24 hours
0.0062037	Use of a mechanical ventilation device or registered oxygen saturation below 93% in the last 24 hours
0.0035883	Age group, 40-59 years
0.0032147	Maximum respiratory rate higher than 24 irpm in the last 24 hours
0.0012283	Days after the removal of a non-invasive mechanical ventilation device
0.0011445	Use of a non-invasive mechanical ventilation device in the last 24 hours
0.0011158	Length of stay in ICU beds (days)
0.0006577	Age group, 1 to ≥40 years
0.0001957	Length of stay in semi-ICU beds (days)
0.0000010	Days after the removal of invasive mechanical ventilation device
0.0000000	Heart rate above 125 bpm in the last 24 hours
0.0000000	Registered oxygen saturation below 93% in the last 24 hours
0.0000000	Prescription of vasoactive drugs in the last 24 hours
0.0000000	Hemodialysis patient
0.0000000	Prescription of vasoactive drugs or heart rate above 125 bpm in the last 24 hours

ICU: intensive care unit.

The Random Forest Model, which was proposed to estimate the total daily LOS for all hospitalized patients, including the detailed criteria above, achieved the following metrics: R² = 0.69, MAE = 2.93 days, and RMSE = 4.09 days.

To deliver outputs that the hospital managers could directly interpret, the results of the estimators were combined so that their composition established a predictive model capable of estimating the number of patients hospitalized in the ICU and general ward per day for the subsequent 14 days, with daily updates and performance monitoring.

Model 2: in the second model, the historical series explored in the analysis were pre-processed following two main operations-the calculation of weekly moving averages to smooth their daily variations, and standardization of the model using the z-score to favor visualization and allow the intuition of an optimal correlation value for pairs of series.

**Figure 1:**
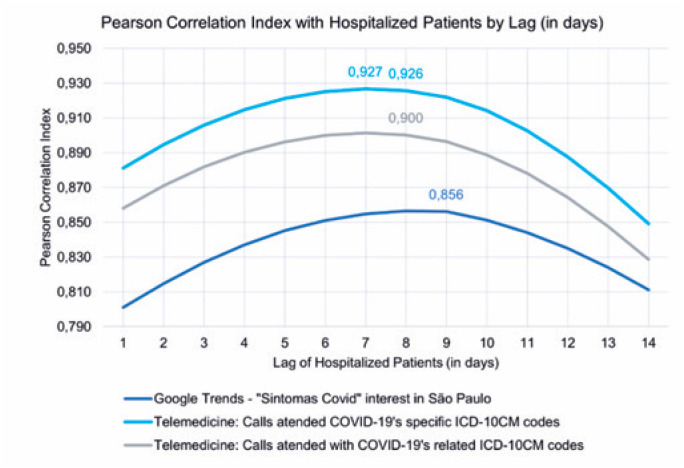
Pearson Correlation Index calculated between all available time series and the lagged series of daily hospitalized patients

The three time series were plotted: i) Telemedicine: number of callers diagnosed with COVID-19 specific coding (ICD-10-CM U07.1); number of callers with COVID-19-related ICD-10-CM codes (specific coding, symptoms and chapters related to respiratory diseases); and ii) Google Trends: daily interest scores for the search term "sintomas COVID-19" in São Paulo. The time series of hospitalized patients lagged by 1–14 days, and for each combination of series, the Pearson Correlation Index was calculated iteratively. The results are presented in figure 1.

The number of telemedicine visits by patients coded with a COVID-19 diagnosis (ICD-10-CM U07.1) was highlighted as the best predictor of hospitalized patients. Once peak values differed by >0.001 for delays of seven and eight days, the delay that had the greatest anticipation power for the proposed model (8 days) was chosen.

In a scenario in which telemedicine data were unavailable, Google Trends interest scores for the terms related to symptoms appeared as a predictor, resulting in a Pearson Correlation Index of 0.895 when lagging the hospitalized patients’ series by 9 days. The final model consisted of a time series linear regression comprising the predictors to estimate the number of hospitalized patients based on the telemedicine series.^(17)^

### Performance monitoring

The results of the two models were followed for 365 days (between May 20, 2021, and May 20, 2022), considering that the first model has predictions for hospitalized patients segmented by department (general ward and ICU) over a 14-day interval and that the second model using the number of telemedicine visits predicts the overall number of hospitalized patients over an 8-day interval.

When comparing models, the evaluation of their performance indicators is limited by the lowest forecast horizon of 8 days. Computing the average error values, including different time windows, could benefit the model with the smallest forecast horizon, as larger residuals tend to be observed as we move away from the projection date.

**Figure 2:**
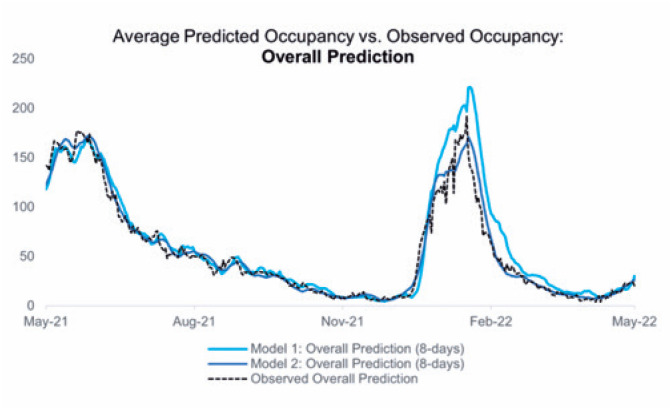
Comparison between the daily number of hospitalized patients and the mean values predicted by the described models

**Figure 3:**
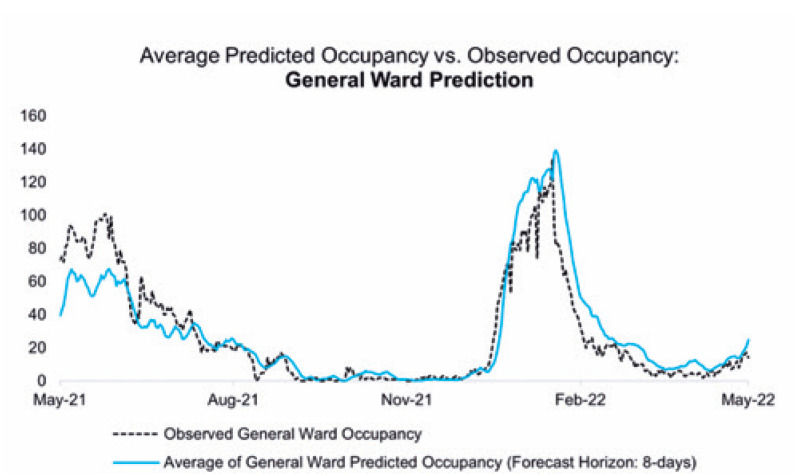
Comparison between the daily number of hospitalized patients in the general ward and the mean values predicted by Model 1

**Figure 4:**
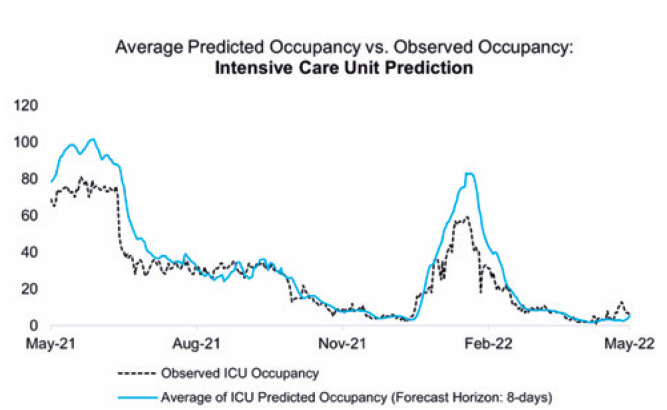
Comparison between the daily number of hospitalized patients in the intensive care unit and the mean values predicted by Model 1

A comparison between the averages of the predicted and observed values by department is illustrated in figures 2, 3, and 4.

**Figure 5:**
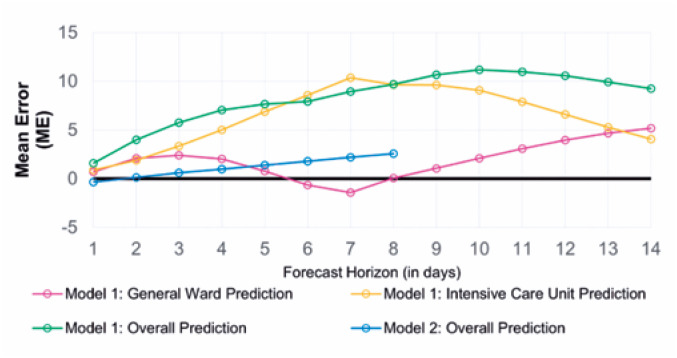
Prediction performance monitoring. Calculated mean error for the proposed models by forecast horizon (days)

**Figure 6:**
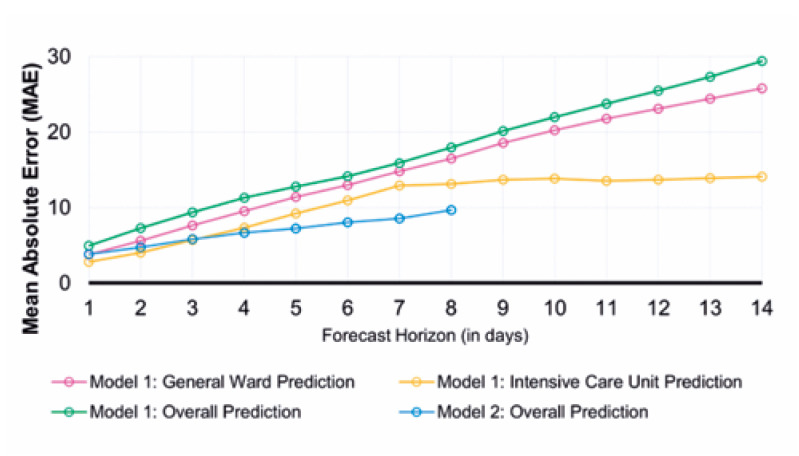
Prediction performance monitoring. Calculated mean absolute error for the proposed models by forecast horizon (days)

**Figure 7:**
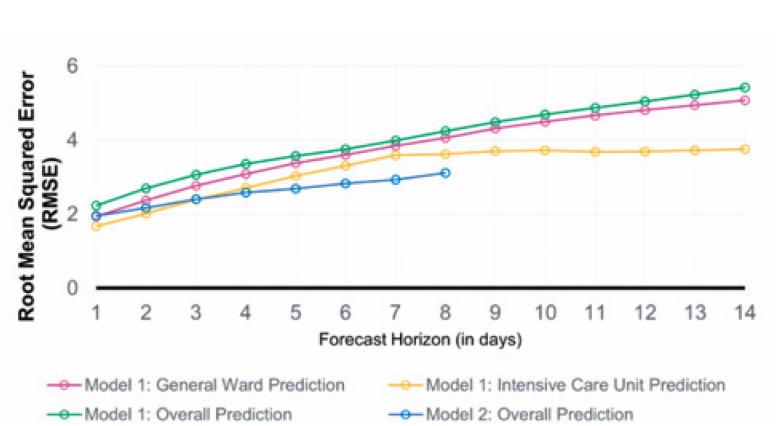
Prediction performance monitoring. Calculated root mean squared error for the proposed models by forecast horizon (days)

The adherence of the values predicted by the proposed models to the numbers observed during these 365 days can be reinforced by the performance indicators detailed in the Methods section, which also allowed the identification of the strengths and opportunities in both models. Figures 5, 6, and 7 present the ME, MAE, and RMSE values as the forecast horizon increases (the illustrated indicators are accessible in table 1S form in Supplementary Material).

The ME statistic allows the identification of possible biases in the proposed models. A positive value indicates that the predictions obtained using the referred model are consistently higher than the observed value; in this case, the number of hospitalized patients is overestimated. All predictions have a positive bias, and the model that processes clinical data overestimates the number of hospitalized patients the most, mainly because of error propagation involving the prediction of patient occupancy in the ICU.

The MAE accuracy measure, which is a measure minimized by the median, allows the identification of the models that can most accurately estimate the daily number of hospitalized patients. Despite the ME values tending to zero for the predictions of hospitalizations in the general ward for the first model in 10-day forecast horizons, rejecting the presence of bias in the set of estimations, the result is obtained through predictions with greater residues. As a complementary approach, the RMSE highlights the model that provides the best estimates for the average number of cases in a given period.

The analysis of all three statistics showed that the model built with telemedicine data outperformed the first model when considering the predictions made for the total number of hospitalized patients, also highlighting its capability of maintaining errors below 10 beds for the integrity of its forecast horizon, as evidenced by the MAE.

The first model shows significant deterioration over a period >3 days; however, its ability to distinguish between the classifications of occupied beds sets a gain for resource planning, even though higher reliability indices are limited to short-term predictions.

## DISCUSSION

The developed models resulted in reliable estimations of the expected number of COVID-19 hospitalizations at HIAE, which were updated daily and used by HIAE managers to define the allocation of beds to COVID-19 and non-COVID-19 patients, mainly by adjusting the volume of elective surgeries that the hospital would be able to perform in the subsequent 7 days, the hiring of qualified professionals to meet the expected demand, and the proper sizing of its supplies.

Daily capacity adjustments were performed at HIAE during the worst days of the pandemic based on the proposed telemedicine model and were also used by the hospital to prepare for the Delta and Omicron phases of increase in the observed number of hospitalized patients.

The development and implementation of a data-driven tool to support the decision-making process in a hospital management environment showed that at an atypical moment of great concern with the evolution of the COVID-19 pandemic, maturity in data management, quality, security, and analysis allows a health institution to benefit from the information generated during day-to-day operations.

The detailed approach assessed the inclusion of clinical variables and hospital data aimed at delivering estimations that accurately capture the nuances of demand behavior, trends of increases and decreases in new cases and hospitalizations, and the epidemiological profile of hospitalized patients.^(18)^ As an alternative to population models, which became popular throughout the COVID-19 pandemic, and aiming for estimates that comprise geographic regions in their entirety, models can be developed by applying algorithms oriented to the projection of time series, such as ARIMA^(19)^ and Convolutional Neural Networks.

The isolation of the hospital context and its variables implies a trade-off, given that the variables that can explain the remaining variance of the studied phenomenon may be unavailable as the pandemic context evolves dynamically. Among these caveats are the emergence of new variants, vaccines and vaccine coverage, effective medications, mask usage, isolation rates, and the guidelines adopted in care.

The validation of predictions of this nature encourages the creation of new models for other pathologies, exploring correlations of events before hospitalizations, and studying patterns of care involving a diagnosed patient and their clinical evolution. It also highlights the importance of joining forces, between different areas of the same institution and between various institutions, sharing complementary data, and improving the use of data for decision-making.

## CONCLUSION

The model that estimates the number of COVID-19 hospitalizations in a private not-for-profit hospital based on telemedicine could accurately anticipate the increase and decrease in the volume of patients with a lag of 8 days, demonstrating its usefulness for the effectivemanagement of beds and general resources for caring for patients with COVID-19. The readiness to provide data regarding the volume of care, hospitalizations, patient characteristics, and clinical interventions can help identify patterns to understand the pathology better and provide more accurate decisions regarding the allocation of resources.

## Table 1S Performance metrics as a function of the forecast horizon

**Table:**

	Mean Error				Mean absolute error				Root mean squared error			
Forecast horizon (in days)	Model 1: General ward prediction	Model 1: Intensive care unit prediction	Model 1: Overall prediction	Model 2: Overall prediction	Model 1: General ward prediction	Model 1: Intensive care unit prediction	Model 1: Overall prediction	Model 2: Overall prediction	Model 1: General ward prediction	Model 1: Intensive care unit prediction	Model 1: Overall prediction	Model 2: Overall prediction
1	0,719	0,865	1,584	-0,343	3,758	2,792	4,961	3,808	1,939	1,671	2,227	1,951
2	2,078	1,906	3,984	0,117	5,605	4,052	7,288	4,725	2,368	2,013	2,700	2,174
3	2,384	3,361	5,745	0,587	7,642	5,730	9,387	5,792	2,764	2,394	3,064	2,407
4	2,039	4,992	7,031	0,979	9,519	7,356	11,286	6,694	3,085	2,712	3,359	2,587
5	0,771	6,878	7,649	1,397	11,384	9,226	12,787	7,236	3,374	3,037	3,576	2,690
6	-0,652	8,564	7,912	1,777	12,969	10,927	14,125	8,026	3,601	3,306	3,758	2,833
7	-1,426	10,356	8,930	2,182	14,787	12,938	15,901	8,566	3,845	3,597	3,988	2,927
8	0,060	9,639	9,699	2,566	16,491	13,125	17,984	9,673	4,061	3,623	4,241	3,110
9	1,060	9,616	10,675		18,587	13,683	20,130		4,311	3,699	4,487	
10	2,088	9,088	11,177		20,265	13,847	21,951		4,502	3,721	4,685	
11	3,083	7,899	10,982		21,758	13,561	23,771		4,665	3,683	4,876	
12	3,969	6,608	10,577		23,117	13,668	25,496		4,808	3,697	5,049	
13	4,657	5,273	9,930		24,418	13,891	27,296		4,941	3,727	5,225	
14	5,177	4,062	9,239		25,769	14,109	29,410		5,076	3,756	5,423	

The results are expressed as the mean error, mean absolute error, and root mean squared error calculated for the predictions.
